# Cocaine and Sleep: Early Abstinence

**DOI:** 10.1100/tsw.2007.209

**Published:** 2007-11-02

**Authors:** Peter T. Morgan, Robert T. Malison

**Affiliations:** Department of Psychiatry, Yale University School of Medicine, New Haven, CT, USA

**Keywords:** cocaine, sleep, insomnia, abstinence, treatment, GABA

## Abstract

Compulsive cocaine use is associated with a profound dysregulation of sleep. Perhaps the result of chronic use, a significant deterioration in sleep is apparent over the first 3 weeks of abstinence, with no indication of recovery. Interestingly, the diminished sleep is not accompanied by subjective reports of poor or worsening sleep. Rather, subjective reports actually *improve* over abstinence, while sleep-related cognitive performance declines. A mechanistic understanding of the apparent difference in objective and subjective measures is currently lacking. Here we review the relevant literature on cocaine use and sleep, and discuss the possible relevance of this sleep disturbance in relationship to the underlying disorder and its treatment.

## INTRODUCTION

Cocaine dependence is one of many psychiatric illnesses in which sleep problems are common[1]. Emerging evidence suggests that the sleep problems associated with psychiatric illnesses may be more than just symptomatic consequences of the underlying illness[1,2,3,4,5,6,7]. Rather, the sleep disturbances associated with psychiatric illness may be integral to the underlying disease process. If so, appropriate treatment of the sleep disturbance associated with psychiatric illness would be much more than symptom management, as it may be essential to promote recovery fully.

For cocaine dependence, this possibility may be particularly important. There is currently no FDA-approved medication for the treatment of cocaine dependence despite many efforts toward this end, so the identification of a novel target for medicinal therapy could have far-reaching implications. Furthermore, recent evidence suggests that the sleep disturbance associated with cocaine dependence persists through at least 3 weeks of abstinence and is associated with sleep-related cognitive deficits[8,9,10,11]. The duration of this disturbance and the apparent functional consequences suggest that the sleep disturbance associated with cocaine dependence may be particularly relevant to relapse to cocaine use.

To use the sleep disturbances associated with cocaine dependence as a target for treatment, the characteristics of these disturbances must be well described. This review summarizes the published polysomnographic sleep studies of persons with cocaine dependence, as well as some studies of self-reported sleep and related symptoms in this population. The results from these studies are compared and discussed, and the possible relevance to treatment is considered.

## POLYSOMNOGRAPHIC (PSG) MEASUREMENT OF SLEEP IN COCAINE DEPENDENCE

From 1992 through the present, six published studies[9,10,12,13,14,15] reported polysomnographic sleep measurement in primarily cocaine-dependent (nonopioid dependent), primarily male subjects. Excluding the most recent study, these studies are limited by the small number of subjects studied[10,12,13,16], the lack of controlled cocaine administration[12,14,15], and/or the lack of specificity for cocaine[14,15]. Despite design differences, many findings from these studies are consistent with regard to the effects of cocaine administration and abstinence on sleep (Table 1). The most consistent finding among these studies is the severely disrupted sleep that is most evident after 11 days of abstinence, or more, without evidence of recovery as far as the 3rd week of abstinence. This disruption is seen as an increase in the time it takes to initiate sleep (increased sleep latency), decreased total time spent asleep, and decreased percentage of time in bed that is spent sleeping (decreased sleep efficiency).

Sleep architectural changes are also seen in response to cocaine and cocaine abstinence. On the first night of sleep following laboratory cocaine administration, there is evidence of REM suppression (REM latency relatively increased, REM sleep time decreased, and/or decreased REM pressure)[8,13,16]. A rebound from this suppression is evident within the first few days of abstinence, with decreased REM latency and increased REM sleep time (along with increased total sleep time). In contrast, laboratory cocaine administration seems to increase slow-wave sleep activity on the first night, but as REM sleep increases in the first few days of abstinence, slow-wave sleep decreases[8,9]. As abstinence progresses into the 3rd week, REM sleep diminishes to very low levels and slow-wave activity is proportionally higher (but overall slow-wave sleep time is still low compared to age-matched normative data). Characteristic examples of how cocaine users' polysomnographically measured sleep change over the course of abstinence are shown in Fig. 1.

## SELF-REPORTED EFFECTS OF COCAINE AND COCAINE ABSTINENCE ON SLEEP

In contrast to PSG measured sleep, self-reported sleep and related measures, such as daytime sleepiness, energy, concentration, and confusion have shown a much different pattern of response to abstinence. Table 2 summarizes results from five studies that measured self-report of sleep and related measures in chronic cocaine users across the first 15 days or more of abstinence[9,10,15,17,18]. One study[9] also reported these measures on the nights and mornings after controlled cocaine administration. Although perhaps not as consistent as the polysomnographically measured sleep data, self-reports of sleep quality and related measures largely improve over the period of abstinence measured in these studies.

The improvement seen in sleep-related measures largely parallels improvements in mood, cocaine craving, and other cocaine withdrawal measures[17]. Most evidence supports monotonic improvements in these measures over abstinence, with some evidence supporting phasic vs. continuous changes. For example, Weddington et al.[17] show a precipitous drop in fatigue over the first 4 days, followed by little change, then another drop at 10 days. It should be noted that Weddington et al.[17] only reported statistical analysis for overall time effects, without comparison of one time point to another, despite several measures showing possible bi- or triphasic response to time abstinent. Morgan et al.[9] found that mental alertness improved significantly at 4 days abstinence relative to earlier in abstinence, but showed no further improvement as abstinence progressed. Other studies (e.g., Coffey et al.[18]) did not measure with sufficient frequency to distinguish phasic vs. continuous changes.

Overall sleep quality self-reported measures, when found to increase, tended to do so continuously over abstinence[9,17], and in Weddington et al.[17] were similar to increases seen in a control group. This raised the possibility that the increases were due to acclimation to a new environment, i.e., the inpatient hospital. However, six of the 12 subjects from Morgan et al.[9] received laboratory cocaine after being in the hospital for 17 days and showed marked drops in self-reported sleep quality on the first 3 days of abstinence after

**TABLE 1**
**Studies with PSG Sleep Measurement**

| | Morgan et al.[9] | Pace-Schott et al.[10] | Johanson et al.[13] | Thompson et al.[14] | Gillin et al.[15] | Watson et al.[16] | Kowatch et al.[12] |
|---|---|---|---|---|---|---|---|
| Number of cocaine-dependent subjects | 12 | 5 | 3 | 7* | 11** | 3 | 3–9*** |
| Laboratory cocaine administration | Yes | Yes | Yes | No | No | Yes | No |
| Controlled abstinence | Yes | Yes | Yes | Yes | Yes | Yes | Yes |
| Number of days of abstinence observed | 17 | 15 | 14 | Up to 14 | ~20 | 3 | Up to 17 |
| Comparison to control or normative data | Yes | No | Yes | Yes | No | No | Yes |
| REM suppression by cocaine | Yes† | No | Yes | Not measured | Not measured | Yes | Not measured |
| REM rebound in early abstinence | Yes | No | Yes | Yes | Yes | Yes | Yes |
| REM decline in later abstinence | Yes | Yes†† | Yes | Yes | Yes | Not measured | No |
| Total sleep time rebound in early abstinence | Yes | No | Yes | No | Not reported | Yes | Yes |
| Insomnia-like sleep‡ after 11 days of abstinence or more | Yes | Yes | Yes | Yes | Yes | Not measured | Yes |
| Slow-wave sleep/slow-wave activity effects | Yes | Limited | Not reported | No | Yes | Yes | Yes |
| Increased daytime sleep latency (Multiple Sleep Latency Test) | Not measured | Not measured | Yes | Not measured | Not measured | Not measured | Not measured |

* Reflects two sets of seven subjects: one set measured at 3–10 days abstinent, the other at 11–14 days abstinent. Of the 14 total subjects, five also (three) or exclusively (two) used amphetamine.

** Includes some subjects with amphetamine dependence, or cocaine and amphetamine dependence

*** Data from between three and nine subjects were reported at each of several time points from 2–17 days abstinent.

† REM data from this study reported in Morgan et al.[8].

†† Lack of preservation of REM with declining total sleep time.

‡ Decreased total sleep time, long sleep latency, poor sleep efficiency, and/or prolonged wake time after sleep onset.

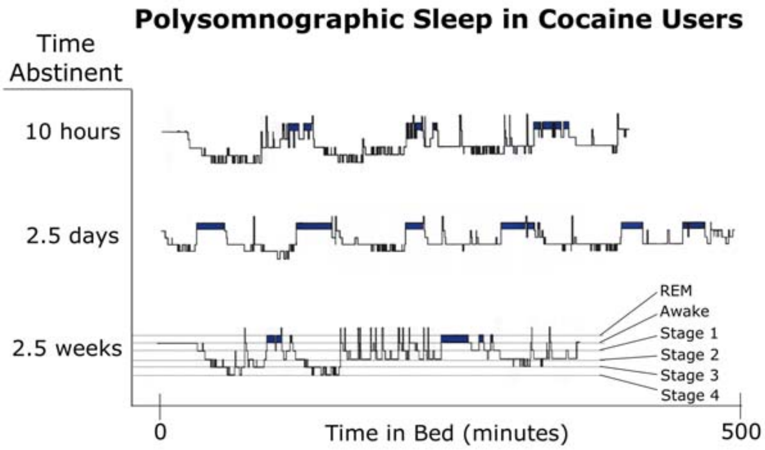

**FIGURE 1**. Characteristic examples of polysomnographically measured sleep at different points along the first 3 weeks of abstinence. On the first night of sleep, *10 h* after administration of 12 i.v. doses of 32 mg cocaine/70 kg body mass, REM sleep is suppressed with decreased REM sleep time and relatively increased REM latency. Slow-wave (stages 3 and 4) sleep is prominent in the beginning of the night. Sleep latency is somewhat increased and total sleep time is decreased. *At 2.5 days abstinent*, a rebound in REM sleep occurs with decreased REM latency and increased REM sleep time. Slow-wave sleep is much less prominent, sleep latency has decreased, and total sleep time has increased. *After 2.5 weeks of abstinence*, sleep is insomnia-like, with increased sleep latency, low sleep efficiency (% time spent asleep while in bed), and decreased total sleep time. REM sleep time is very short despite a REM latency that is decreased compared to healthy subjects, and the proportion of slow-wave sleep has increased relative to 2.5 days abstinent. Excursions of the hypnograms above the REM line indicate movement or other artifacts.

cocaine administration (i.e., after 21 days in the hospital) relative to the few days prior to cocaine administration. This finding leaves open the possibility that the improvement in self-reported sleep with abstinence is related to abstinence itself and not just acclimation to the sleep environment. Overall, there is consistent evidence that there is no decline in self-reported sleep quality as abstinence progresses, and substantial evidence that both perceived sleep quality and related measures, such as fatigue, alertness, and concentration, improve over the first 2–4 weeks of abstinence.

## THE DISCREPANCY BETWEEN PSG AND SELF-REPORTED SLEEP MEASURES

Typical responses to sleep deprivation of the magnitude seen in chronic cocaine users after 2 weeks of abstinence might include sleepiness, fatigue, irritability, poor attentional performance, and increased propensity toward sleep (shortened time to sleep, falling asleep easily during the day)[19,20,21]. Chronic cocaine users, however, are more like insomniacs who have difficulty falling asleep, both at night and during the daytime[13], despite the limited total sleep time. Very much unlike insomniacs, however, chronic cocaine users do not report trouble with their sleep: overall sleep quality and satisfaction with sleep, for example, are comparable to control subjects[17], and as discussed above, either improve or remain stable over abstinence.

It has been hypothesized[9] that the discrepancy between PSG measured and self-reported sleep is due to a dysregulation of the homeostatic sleep drive, the drive toward sleep that builds with increased wakefulness. Such a dysregulation would leave cocaine users with decreased sleepiness (both perception and physiological) despite accumulating deficits secondary to chronic sleep restriction. Alternative explanations considered were that chronic cocaine users simply need less sleep or have more restorative sleep. However, coincident with deteriorating PSG measures of sleep in abstinence are deteriorations of

**TABLE 2**
**Studies with Self-Reported Sleep and/or Related Measures**

| | Morgan et al.[9] | Pace-Schott et al.[10] | Coffey et al.[18] | Gillin et al.[15] | Weddington et al.[17] |
|---|---|---|---|---|---|
| Number of cocaine-dependent subjects | 12 | 5 | 24* | 11** | 12 (+10 control subjects) |
| Substantial (>25%) inclusion of women | No | No | Yes | No | No |
| Laboratory cocaine administration | Yes | Yes | No | No | No |
| Controlled abstinence | Yes | Yes | No | Yes | Yes |
| Number of days of abstinence observed | 17 | 15 | 28 | ~20 | 28 |
| Self-reported sleep quality† | Improved | Unchanged or improved†† | Unchanged | Not measured | Improved (in patients and controls) |
| Self-reported energy or alertness† | Increased | Not measured | Increased | Increased | Unchanged |
| Self-reported fatigue or sleepiness† | Not measured | Not measured | Decreased*** | Decreased | Decreased |
| Self-reported concentration† | Not measured | Not measured | Improved | Not measured | Not measured |
| Self-reported confusion† | Not measured | Not measured | Decreased | Decreased | Decreased |

* Of the 24 completers (out of 82 enrolled), 12 subjects were dependent on both cocaine and alcohol.

** Includes some subjects with amphetamine dependence, or cocaine and amphetamine dependence.

*** Statistical trend.

† Change with progressive abstinence.

†† Depending on measure used.

sleep-related cognitive performance, such as attentional measures[9,11] and sleep-dependent learning[8,9]. These findings strongly suggest that the apparent deficiencies in chronic cocaine users' sleep are indeed deficiencies, with functional consequences that could affect treatment retention[22] and propensity to relapse[23,24]. Intriguing in this "occult insomnia"[9] is the possibility that the cognitive deficits associated with sleep restriction may be separable from the perception of fatigue and sleepiness.

## GENDER CONSIDERATIONS

With little exception, research discussed here has focused largely or entirely on male cocaine users. The one study that had a substantial proportion of female subjects[18] reported no gender differences in self-reported measures of sleep and sleep-related variables across abstinence. However, as yet unpublished data[25] suggest that male and female cocaine users may have markedly different physiologically measured sleep responses to abstinence. In this study, women did not show a deterioration in sleep with abstinence, did not show sleep-dependent learning deficits, and did not differ from control subjects in total sleep time, sleep efficiency, or sleep-dependent learning at 3 weeks of abstinence (whereas men showed all of the above). As the women in this study were all in the luteal phase at 3 weeks abstinence, it is possible that progesterone or its metabolite allopregnanolone could be influencing sleep through $GABA_A$ receptor mechanisms.

## MECHANISMS OF COCAINE EFFECTS ON SLEEP

There are only limited data that directly examine the relationship between specific neurotransmitter systems and the effects of chronic cocaine use on sleep. Gillin et al.[15] found that the dopamine D2 receptor agonist lisuride (that also has serotonergic effects) reduced the REM sleep rebound seen in early abstinence from cocaine. This finding suggests that the increase in synaptic dopamine (and perhaps serotonin) due to cocaine could cause the REM inhibition seen with recent use. However, the GABA system is both of critical importance to sleep, is closely linked to dopamine neurotransmission and cocaine dependence (with evidence that GABA receptor agonists modulate aspects of cocaine use behavior), and alterations in the GABA system can have effects similar to those observed during cocaine abstinence[26,27,28,29,30,31,32,33,34,35,36]. Hence, in the absence of more specific data, we hypothesize that the sleep disturbance seen in abstinence from chronic cocaine use is most directly mediated by alterations in GABA signaling that are the result of adaptation to repeated overstimulation of monoaminergic pathways.

## POSSIBLE TREATMENT IMPLICATIONS

The presence of an ongoing and functional disruption of sleep in chronic cocaine users raises the possibility of treatment of cocaine dependence through a sleep-affecting medication. The characteristic effects of cocaine and cocaine abstinence on sleep architecture should be considered in such treatment considerations. The low levels of REM sleep present in abstinence beyond the initial rebound caution against a REM suppressing agent. Although cocaine itself is effective in temporarily reversing the sleep time and REM deficits that develop in the $2^{nd}$ week of abstinence, it is only in the withdrawal from cocaine (after at least 24 h of abstinence) that this temporary reversal is achieved. Hence, from the standpoint of treating the sleep deficit of cocaine dependence, long-acting dopamine agonists (replacement therapy) given alone should not be effective, but perhaps worsen or at least sustain the problem. This may be particularly true for the typical “binge” user of cocaine, where periods of heavy use are followed by several days of nonuse. In addressing the shortened total sleep time, an agent that promotes slow-wave sleep and perhaps aids cognitive recovery from sleep restriction (e.g., tiagabine[37]) may be more helpful than an agent that extends sleep only through promoting stage 2 sleep (e.g., benzodiazepines). Notably, promising candidate pharmacotherapies for cocaine addiction either have effects on sleep or daytime wakefulness/cognition: gamma-vinyl GABA[35], tiagabine[33,34], and topiramate[38] may alter sleep through GABAergic mechanisms; modafinil[39] improves vigilance and appears to alter the homeostatic sleep/wake drive[40]. An agent not yet tested in cocaine dependence, gaboxadol (a modulator of largely extrasynaptic $GABA_A$ receptors), is particularly interesting as it both promotes slow-wave sleep and lengthens total sleep time[41,42]. The intriguing possibility that luteal-phase women are protected against the observed sleep disruption suggests that a natural sex hormone (e.g., progesterone) could play a role in treating this aspect of cocaine abstinence

## ACKNOWLEDGMENTS

This work was supported by NIH grants K12RR17594 (PTM), R01 DA15857 and K02 DA00397 (RTM), the Yale General Clinical Research Center (funded by NIH/NCRR/GCRC Program Grant M01-RR00125), and the Connecticut Department of Mental Health and Addiction Services (DMHAS).
